# Awake prone positioning in a patient with respiratory impairment due to subarachnoid hemorrhage: a case report

**DOI:** 10.1186/s13256-025-05311-1

**Published:** 2025-05-28

**Authors:** Giorgia Pacchiarini, Federico Geraldini, Alessandro De Cassai, Giulia Aviani Fulvio, Annalisa Boscolo, Francesco Zarantonello, Paolo Navalesi, Marina Munari

**Affiliations:** 1 Department of Medicine, University of Padua, Padua, Italy; 2 Sant’Antonio Anesthesia and Intensive Care Unit, University-Hospital of Padua, Padua, Italy; 3 Anesthesia and Intensive Care Unit, University-Hospital of Padua, Padua, Italy; 4 Department of Cardiac, Thoracic, Vascular Sciences and Public Health, University of Padua, Padua, Italy

**Keywords:** Pulmonary gas exchange, Pronation, Respiratory insufficiency, Respiratory distress syndrome, Subarachnoid hemorrhage

## Abstract

**Background:**

Prone positioning has been shown to be an effective rescue strategy in severe acute respiratory distress syndrome and was widely used during the coronavirus disease 2019 pandemic, both in mechanically ventilated and in awake patients. Subarachnoid hemorrhage is often associated with respiratory failure. Prone positioning has been used in brain-injured patients, but concerns relating to neurological complications from intracranial hypertension still remain.

**Case presentation:**

We report the case of a 59-year-old Italian patient with subarachnoid hemorrhage who safely underwent awake prone positioning after deterioration of respiratory function.

**Conclusions:**

In this report, we show that awake pronation is possible in patients with subarachnoid hemorrhage. However, careful monitoring of Intracranial pressure and clinical examination may be the keys to successful application of this procedure.

## Background

Prone positioning has a proven role in reducing morbidity and mortality and improving outcome in patients with acute respiratory distress syndrome (ARDS) [1, 2]. During the coronavirus disease 2019 (COVID-19) pandemic, prone positioning was often adopted as a strategy to improve patient recovery and was not exclusive to the intensive care unit (ICU) setting. In fact, pronation was demonstrated as feasible and potentially effective also in awake patients requiring oxygen supplementation, improving blood oxygenation and respiratory function [3–5].

Aneurysmal subarachnoid hemorrhage (SAH) is a disease with high disability and mortality rates [6, 7]. In addition to the direct effects of the initial hemorrhage and secondary neurological insults, SAH predisposes to systemic complications, including respiratory failure due to pulmonary edema, aspiration pneumonia, and ARDS. These systemic conditions can have an impact on outcome and increase hospital length of stay [7–9].

Prone position has been adopted as a rescue strategy in sedated patients with brain injury and associated refractory respiratory failure [10–13], and its use has been proposed also for mechanically ventilated patients with SAH [14]. Given the above, we hypothesized that awake prone positioning could be feasible and effective in selected patients suffering from SAH and associated worsening respiratory status.

## Case presentation

**Fig. 1:**
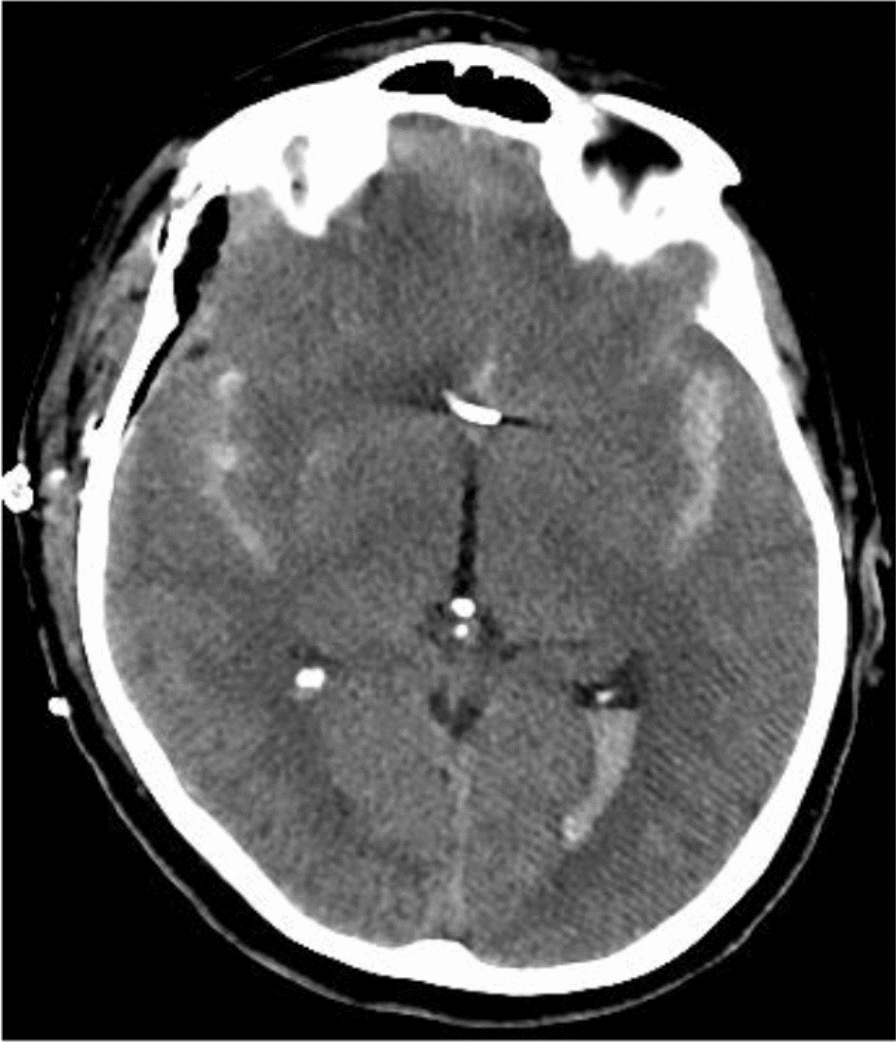
Computed tomography scan showing subarachnoid hemorrhage and the clip on the aneurysm

Our patient was a 59-year-old Italian man, weighing 85 kg, a former smoker with no relevant previous medical history. The patient presented with severe headache and neck pain, followed by temporary loss of consciousness and associated head trauma. After the initial event, he reached the Emergency Room independently, with a World Federation Of Neurosurgical Societies (WFNS) grading scale of 1. The initial cerebral computed tomography (CT) scan showed a modified Fisher grade III SAH (Fig. 1). Angio CT study was subsequently performed, confirming the presence of a 4-mm aneurysm of the anterior communicating artery.

The first treatment option was endovascular coiling, but the attempt was unsuccessful. The patient was therefore transferred to the operating room for surgical clipping of the aneurysm, performed under general anesthesia. The operation was uneventful, and the patient was transferred to the ICU for postoperative monitoring. Intravenous infusion of nimodipine was started to prevent vasospasm, and the patient’s clinical progression was monitored through regular clinical examinations, daily bedside transcranial Doppler (TCD) assessments, and serial cerebral CT scans. The CT scans were performed every 3 days on average, starting after the 24-h post-intervention scan, for a total of 12 days. He rapidly started weaning from sedation and ventilation and was successfully extubated 2 days after the initial hemorrhage. Hemodynamic support with norepinephrine was necessary in the first phases and was discontinued with the de-escalation of sedative drugs.

During the following days, he showed a progressive deterioration of respiratory function, with worsening oxygenation and respiratory fatigue. Subsequent chest radiography rays showed consolidation of the lower right lobe and pleural effusion with fever, highly suggestive of pneumonia. Antibiotic therapy with amoxicillin–clavulanate was started. Four days after the initial event, the patient presented with respiratory distress and involvement of accessory muscles. Given the good neurological function after the SAH, we decided to attempt awake prone positioning. There were no complications during the maneuver, and the patient underwent two cycles of continuous prone positioning (8 and 4 hours, respectively), both interrupted for patient intolerance. Neurological status was evaluated clinically, and TCD was used daily to monitor for vasospasm during the cycles, showing normal velocities with a Lindegaard ratio between 1.9 and 2.1. Despite the beneficial effect of prone positioning on gas exchange and respiratory function, the patient refused to perform a third cycle of awake prone positioning. After a failed noninvasive ventilation trial, awake fiberoptic intubation under local anesthesia was performed, and the patient was sedated with propofol; the physician decided to use fiberoptic intubation rather than traditional general anesthesia induction, weighing the risk of possible rise in systolic pressure in a patient with a secured cerebral aneurysm against the potential dangers of hypoxia and hypercapnia that could arise from prolonged intubation during anesthesia induction. After sedation, hemodynamic support with norepinephrine was necessary to maintain adequate cerebral perfusion pressure values. Five days after the initial event, a third pronation cycle was performed under general anesthesia, lasting a total of 16 hours; during this cycle, clinical monitoring was restricted owing to prone position and deep sedation, while TCD was used to confirm the absence of vasospasm and to estimate intracranial pressure noninvasively. Upon restoration of supine position, the patient showed a temporary improvement in gas exchange and X-ray findings (Table 1).

**Table 1:** Gas exchange during the awake supine/pronation cycle

Position	Ventilation	O_2_	pH	PaO_2_ (mmHg)	PCO_2_ (mmHg)
Supine	Spontaneous	8 l (mask)	7.46	81.1	32.5
Prone first cycle	Spontaneous	8 l (mask)	7.46	149.1	29.8
Supine	Spontaneous	8 l (mask)	7.42	77.4	30.0
Prone second cycle	Spontaneous	8 l (mask)	7.44	95.9	30.7
Supine	Spontaneous	8 l (mask)	7.41	73.3	35.9
Prone third cycle	Mechanically ventilated	FiO_2_ 0.7	7.53	129.8	34.5

A fourth cycle was therefore started, lasting 12 hours, using TCD to monitor vasospasm and noninvasive intracranial pressure (ICP). With negative blood and pulmonary cultures, antibiotic therapy was empirically modified owing to absence of clinical benefit, shifting from amoxicillin–clavulanate to ceftazidime, levofloxacin, and linezolid. After the pronation cycles, the respiratory status showed progressive improvement, and weaning from sedation and ventilatory support was soon started.

From a neurological point of view, the patient demonstrated phases of diminished level of consciousness and the serial CT scans highlighted the development of hydrocephalus with transependymal edema 19 days after admission. Twenty days after admission, a ventriculo-peritoneal shunt was performed. Given the patient’s impaired ability to cough, a percutaneous tracheostomy was performed 26 days after admission. Weaning from ventilatory support proceeded regularly and was completed 5 days later.

Forty days after admission, the patient was finally transferred from the ICU to the neurosurgical ward, and a week later he was discharged to the rehabilitation unit.

At the 3-month follow-up visit, the patient had a modified Rankin score of 1; he had no significant disability and was able to carry out all usual duties and activities. During the follow-up visit, he signed the written informed consent for this report to be published.

## Discussion and conclusion

In this case report, we propose awake prone positioning as a rescue strategy to avoid intubation in a patient experiencing respiratory insufficiency following aneurysmal SAH.

Pulmonary complications are the most common nonneurological cause of death in patients with SAH, and acute respiratory distress syndrome is the most frequently encountered [15]; studies have shown that mortality in this subgroup of patients can increase by as much as 40% [16]. However, other than pulmonary complications, vasospasm and delayed cerebral ischemia are also significant in affecting outcome in patients with SAH, and early recognition and treatment are essential in the management of these patients [6].

The cause of impaired respiratory exchange in patients with SAH is complex and multifactorial. On one hand, brain-injured patients may suffer from swallowing disorders, making them prone to aspiration pneumonia [17]. On the other hand, brain injury is closely linked to lung dysfunction due to abnormal immune responses, catecholamine release, and cytokine storms. These factors can increase pulmonary vascular pressure and activate the lung’s immune system, heightening its susceptibility to secondary damage [18].

Therapeutic prone position was first proposed over 45 years ago for patients with impaired respiratory exchange, and its beneficial effects have been demonstrated in numerous studies [19], and more recently pronation was demonstrated feasible and potentially effective also in awake patients suffering from ARDS [3–5].

However, neurocritical patients are at high risk of ICP [20] as the net effect of the hemorrhage and cerebral edema (primary brain injury) causes a raise in ICP that can determine ischemia and worsen cerebral edema, leading to secondary brain injury. For this reason, avoiding factors that can negatively influence an increase in ICP is of paramount importance. Among them, patient positioning is one of the cornerstones in preventing ICP and worsening of the clinical condition. In these patients, optimizing patient position includes raising the head of the bed to 30° and careful mobilization.

For these reasons, prone positioning is not usually considered in this patient population as a therapeutic option in case of ARDS in patients with SAH. However, data on mechanically ventilated populations with neurological injury show that the benefits of prone positioning outweigh the expected adverse effect of the position as the cerebral blood flow seems not to be altered [13, 14] even if ICP elevations and reduction in cerebral perfusion could occur during this maneuver. It has been proven that this complication could be managed and adequately treated without harm to the patient under the mandatory condition that intracranial pressure, cerebral perfusion pressure, and brain tissue oxygenation be monitored [21]. Given the above and since pronation can be effective in improving patient outcome in case of severe ARDS, it is our opinion that it should be considered when patient safety can be guaranteed, even in awake patients.

Monitoring of ICP can be achieved by both invasive and noninvasive techniques [20–22]. Invasive monitoring can be performed with intraparenchymal devices or intraventricular catheters and allows a more precise and reliable measure of ICP. Noninvasive ICP monitoring may be useful either as a complementary tool or to decide whether to initiate invasive monitoring [20]. The noninvasive recommended techniques include brain CT, brain MRI, TCD, and optic nerve sheath diameter [20, 22–24]. All these diagnostic options can be used in combination with the clinical examination, which remains essential. Clinical tools that have been studied to monitor changes in ICP are the Glasgow Coma Scale, motor posturing, and pupillary dilation. However, none of these tools showed a strong reliability in individuating increased ICP if used alone [22]. A combination of many different noninvasive diagnostic tools allows a more comprehensive evaluation of the patient and can help identify increases in ICP, preventing SBI.

In this case, monitoring was performed initially with a clinical examination, as the patient was awake for the pronation cycles. Subsequently, bedside techniques such as TCD and serial CT scans ensured adequate monitoring of the neurological condition. In case of suspected ICP increase, invasive monitoring would have been appropriate.

## Conclusion

Awake prone positioning appears to be feasible and could represent a valid option for the treatment of respiratory failure in patients with SAH. Further study and validation with randomized clinical trials on an adequate sample of patients is necessary, especially to better define the safety and potential benefit of this technique in this population of patients.

## Data Availability

Not applicable.

## Abbreviations

ARDS: Acute respiratory distress syndrome
CT: Computed tomography
ICU: Intensive care unit
ICP: Intracranial pressure
SBI: Secondary brain injury
SAH: Subarachnoid hemorrhage
TCD: Transcranial Doppler
